# Antimicrobial activity of bovine lactoferrin against *Gardnerella* species clinical isolates

**DOI:** 10.3389/fmicb.2022.1000822

**Published:** 2022-09-08

**Authors:** Alessandra Pino, Tommaso Mazza, Maura-Ann H. Matthews, Stefano Castellana, Cinzia Caggia, Cinzia L. Randazzo, Gary A. Gelbfish

**Affiliations:** ^1^Department of Agricultural, Food and Environment, University of Catania, Catania, Italy; ^2^ProBioEtna s.r.l., Spin-off of University of Catania, Catania, Italy; ^3^Bioinformatics Unit, Fondazione IRCCS Casa Sollievo della Sofferenza, San Giovanni Rotondo, Italy; ^4^Metrodora Therapeutics LLC, Brooklyn, NY, United States; ^5^Department of Surgery, Mount Sinai School of Medicine, New York, NY, United States

**Keywords:** bovine lactoferrin, iron-binding glycoprotein, antimicrobial activity, synergistic activity, *Gardnerella* spp.

## Abstract

*Gardnerella* species play a key role in the development and recurrence of Bacterial Vaginosis (BV), a common imbalance of the vaginal microbiota. Because of the high rates of BV recurrence reported after treatment with standard of care antibiotics, as well as the emergence of antibiotic-resistant BV, the development of alternative treatment approaches is needed. Bovine lactoferrin, a well studied iron-binding glycoprotein with selective antimicrobial activity, may ameliorate vaginal dysbiosis either alone or in combination with antibiotics. The present study evaluated the antimicrobial resistance/susceptibility profile of seventy-one presumptive *G. vaginalis* clinical isolates to metronidazole and clindamycin. In addition, the *in vitro* antimicrobial activity of Metrodora Therapeutics bovine Lactoferrin (MTbLF) against the tested clinical isolates, both alone and in combination with metronidazole and clindamycin, was in depth evaluated using defined-iron culture conditions. All 71 presumptive *G. vaginalis* clinical isolates exhibited resistance to metronidazole, with MIC values greater than 256 μg/ml. Different susceptibility profiles were detected for clindamycin. In detail, the vast majority of the tested strains (45%), exhibiting MIC lower than 2 μg/ml, were considered sensitive; 18 strains (25%) with MIC higher or equal to 8 μg/ml, were classified as resistant, whereas the remaining 21 (30%) were classified as intermediate. MTbLF was tested in culture medium at different concentrations (32, 16, 8, 4, 2, 1, and 0.5 mg/ml) showing ability to inhibit the growth of the tested presumptive *G. vaginalis* clinical isolates, including those metronidazole-resistant, in a dose-dependent and not in a strain-dependent manner. MTbLF, at concentrations ranging from 32 to 8 mg/ml, exerted a statistically different antimicrobial activity compared with lower concentrations (4, 2, 1, and 0.5 mg/ml). A synergistic effect between MTbLF (8 and 4 mg/ml) and clindamycin was revealed for all the tested strains. When tested in the absence of other sources of iron, MTbLF did not support the growth of the tested presumptive *G. vaginalis* clinical isolates. Bovine lactoferrin may be a potential candidate to treat *Gardnerella* species infection.

## Introduction

*Gardnerella vaginalis* is one of the etiological factors responsible for the onset of Bacterial Vaginosis (BV), a common bacterial vaginal infection in women of reproductive age (Muzny et al., 2019; Rosca et al., 2019; Morrill et al., 2020; Vitale et al., 2021). BV is a polymicrobial disorder associated to taxon-rich and diverse bacterial communities, including not only anaerobic microorganisms but also other abnormal subtypes (Morrill et al., 2020; Vitale et al., 2021). *Gardnerella vaginalis* was first isolated by Leopold (1953) from urine of men with prostatitis and cervical swabs of women with cervicitis, including couples. It was described as a micro-aerophilic Gram-negative bacterium with haemolytic activity on defibrinated rabbit blood agar. Several studies highlighted the pivotal role of *G. vaginalis* in BV occurrence and relapse (Schwebke et al., 2014; Castro et al., 2015; Hardy et al., 2015; Abdelmaksoud et al., 2017; Jung et al., 2017). In fact, it is well demonstrated that, compared to other BV-associated species, *G. vaginalis* exhibits higher virulence potential and adhesion capacities along with cytotoxic effect and ability to form biofilm (Patterson et al., 2010; Machado et al., 2013; Alves et al., 2014). *G. vaginalis*, starting the vaginal epithelium colonization, serves as a scaffold for other BV-associated bacteria, such as *Atopobium vaginae*, reclassified as *Fannyhessea vaginae* (Swidsinski et al., 2005, 2008; Castro et al., 2015, 2019; Hardy et al., 2016; Nouioui et al., 2018). Recently, the genetic heterogeneity and taxonomic diversity among the *Gardnerella* species were revised by Castro and co-workers (Castro et al., 2020), confirming the inclusion of more species of *Gardnerella*, namely, *Gardnerella leopoldii, Gardnerella piotii*, and *Gardnerella swidsinskii*. Antibiotic administration, the current standard-of-care treatment for BV (Workowski et al., 2021), does not provide sustained cures and greater than 50 percent of women treated with antibiotics experience BV recurrent by 6 months (Sobel et al., 1993; Bradshaw et al., 2006a). Several innovative treatment options such as biofilm-disrupting agents, probiotics or other strategies to modulate the vaginal microbiome, are an active area of research (Bradshaw and Sobel, 2016; Machado et al., 2016).

Pino and co-workers (Pino et al., 2017) demonstrated the beneficial effect of vaginally administered bovine lactoferrin (bLf) as a promising therapeutic approach for BV. The primary mechanism of action of Lf appears to be *via* its ability to sequester iron, with high affinity (Kd 10–20), creating a bacteriostatic iron-depleted environment (Baker and Baker, 2004). While Lf’s iron-binding properties have long been known, Lf has multiple additional activities (Jenssen and Hancock, 2009) and several of these may be useful in treating BV. These include: anti-biofilm activity along with alterations in lectin-dependent bacterial adhesion and quorum-sensing activity, possibly related to iron deprivation (Ammons and Copie, 2013); antimicrobial peptide sequences on Lf’s surface that can interact with lipopolysaccharide (LPS) on the surfaces of gram-negative bacteria and lipoteichoic acid (LTA) on gram-positive bacteria, damaging bacterial cell membranes and potentiating the effectiveness of antibiotics *via* increasing cell permeability (Ulvatne et al., 2001); and anti-inflammatory properties when delivered intravaginally (Vesce et al., 2014; Trentini et al., 2016). Additionally Lf has antiviral (Siciliano et al., 1999) and antifungal (Kuipers et al., 1999) effects. Elevations in endogenous human lactoferrin (hLf) are reported in BV, however, a number of pathogens, including *G. vaginalis* (Jarosik and Land, 2000) *Neisseriaceae* (Biswas et al., 1999) and *Helicobacter pylori* (Dhaenens et al., 1997) have evolved hLF receptors that can acquire iron directly from hLF, the very mediator designed to limit iron supply. The bacterial lactoferrin binding protein B has two different sites for binding hLF, one associated with obtaining iron and the other related to protection against hLF’s antimicrobial peptides (Ostan et al., 2017). Although human and bovine lactoferrin, share some functional properties, they have only a 70% primary sequence homology (Pierce et al., 1991). As recently demonstrated, *G. vaginalis* strains have evolved hLF receptors allowing to acquire iron directly from hLF, whereas they are unable to bind bLF (Jarosik and Land, 2000).

Metrodora Therapeutics bovine Lactoferrin (MTbLF) is in clinical development as a vaginal suppository for the treatment and prevention of recurrence of BV. The present study analyzed the *in vitro* antimicrobial activity of bovine lactoferrin (MTbLF) against presumptive *G. vaginalis* clinical isolates both alone and in combination with the antibiotics commonly used in clinical practice, such as metronidazole and clindamycin.

## Materials and methods

### Isolation and identification of clinical isolates

A total of 71 presumptive *G. vaginalis* isolates (Supplementary Table 1) were recovered from the vagina of BV-positive patients enrolled at the Obstetrics and Gynecology Department, General Hospital G. Rodolico, University of Catania (Italy), and participated in a clinical trial approved by the local ethics committee (Comitato Etico Catania 1, Azienda Ospedaliero-Universitaria “Policlinico-Vittorio Emanuele” Catania, registration number 157/2019/PO; Pino et al., 2021). In detail, the vaginal discharge was collected from the lateral vaginal wall and the posterior vaginal fornix using a sterile synthetic cotton-tipped swab filled with Transystem Amies Medium Clear (Biolife Srl, Milan, Italy) and immediately transferred to the Laboratory of Microbiology, University of Catania, Italy. Vaginal swabs were streaked onto *Gardnerella vaginalis* Selective Medium (GVSM; Oxoid, Italy), and plates were incubated at 35°C for 48 h in an atmosphere containing 7% of carbon dioxide. After incubation, grey/white colonies with a diameter of 0.25–0.5 mm producing beta-haemolysis were randomly selected and Gram stained. Total genomic DNA was isolated from pure cultures using the QIAamp DNA Mini Kit (QIAGEN, Italy). For molecular identification, the primers *G. vaginalis* R (5′ CAG CAA TCT TTT CGC CAA CT 3′) and *G. vaginalis* F (5′ CGC ATC TGC TAA GGA TGT TG 3′) were used according to Knupp de Souza et al. (2016) and Menard et al. (2008). DNA from the type strains *G. vaginalis* ATCC 14018 and ATCC 14019 were used as positive controls.

### Antimicrobial susceptibility testing

The presumptive *G. vaginalis* clinical isolates were tested for resistance or sensitivity to metronidazole and clindamycin following the method described by Schuyler and co-workers (2016). In detail, the tested clinical isolates grown on Nutrient broth (Oxoid, Italy), supplemented with 5% Sheep Blood (ThermoFisher Scientific, Italy), for 48 h under anaerobic conditions, were standardized to 0.5 McFarland using sterile phosphate-buffered saline (PBS) and spread onto GVSM medium. Metronidazole and clindamycin Etest® strips (Liofilchem, Italy) were applied to each plate. After 48 h of incubation in an anaerobic workstation, the MIC was identified as the lowest antibiotic concentration able to inhibit the bacterial cells growth. For both metronidazole and clindamycin, the Etest® strips covered a range of antibiotic concentrations between 0.016 and 256 μg/ml. The presumptive *G. vaginalis* clinical isolates were classified as sensitive (S), intermediate (I), or resistant (R) based on the breakpoints for clindamycin (S: ≤2; I:4; R:≥8) and metronidazole (S: ≤8; I:16; R:≥32) defined for anaerobic bacteria by CLSI (2012, 2016, 2020). *Bacteroides fragilis* ATCC 25285, *Bacteroides thetaiotaomicron* ATCC 29741, *Clostridioides difficile* AT5CC 700,057, and *Eggerthella lenta* ATCC 43055 were used as quality control strains according to CLSI (2020).

### Culture media and growth conditions

A specific Proteose Maltose Dextrose (PMD) basal medium, containing 15 g/l of proteose peptone No. 3 (BD, Switzerland), 10 g/l of maltose (Merck Life Science, Italy), 2 g/l of dextrose (Merck Life Science, Italy), 1 g/l of Na_2_HPO_4_ (Merck Life Science, Italy), and 1 g/l of NaH_2_PO_3_·H_2_O (Merck Life Science, Italy), was used to perform the experiments. The medium was similar to that reported by Jarosik and co-workers (1998). To precisely quantify the iron content, the PMD medium was subjected to Chelex 100 (Biorad, Italy) resin treatment for 4–6 h at room temperature; then, the Chelex resin was removed by filtration. PMD medium was supplemented with divalent cations (magnesium sulfate 0.1 mm; calcium chloride 0.1 mm; zinc chloride 10 μm) and sterilized at 121°C for 12 min after adjusting the pH to 6.8. The PMD high iron medium was obtained by supplementing the PMD basal medium with filter-sterilized human hemoglobin (500 mg/l, Merck, Germany), whereas the PMD-FAC medium was prepared by adding ferric ammonium citrate (FAC; final concentration of 10 μg/ml which corresponds to 2.1 μg elemental iron) to the PMD basal medium.

### Antimicrobial activity of bovine lactoferrin against presumptive *Gardnerella vaginalis* clinical isolates

The antimicrobial activity of GMP manufactured bovine lactoferrin (MTbLF, lot LLL03MAR17B6 manufactured in 2017, 98.7% purity, 15.3% Fe saturation), provided by Metrodora Therapeutics (Brooklyn, NY), was evaluated against the presumptive *G. vaginalis* clinical isolates 5.1, 9.4, 13.6, 14.2, 16.2, and 17.1, which were randomly chosen based on the antibiotic susceptibility data, reported in Table 1. In detail, *G. vaginalis* 5.1, 14.2, and 16.2 strains were classified as clindamycin resistant, showing MIC of 24, 16, and > 256 μg/ml, respectively. *G. vaginalis* 9.4 and 13.6 strains, with MIC values of 0.38 and 0.047 μg/ml, were considered clindamycin sensitive, whereas the *G. vaginalis* 17.1 strain, showing MIC of 4 μg/ml, was categorized as intermediate. All the aforementioned strains were classified as metronidazole resistant.

**Table 1:** Antibiotic susceptibility profile exhibited by the seventy-one (71) presumptive *Gardnerella vaginalis* clinical isolates against clindamycin.

Strain ID code		
Sensitive *n* = 32 (MIC ≤ 2 μg/ml)	Intermediate *n* = 21 (MIC = 4 μg/ml)	Resistant *n* = 18 (MIC ≥ 8 μg/ml)
3.8, 3.15, 3.21, 4.5, 7.2, 7.9, 7.10, 7.21, 9.4*, 10.6, 10.7, 10.9, 10.13, 11.1, 11.6, 11.3, 12.1, 12.7, 12.2, 12.8, 13.6*, 14.5, 14.8, 18.3, 19.6, 20.4, 20.5, 20.2, 26.3, 26.7, 26.9, 27.4	4.2, 4.3, 4.4, 6.1, 6.2, 6.5, 7.4, 7.5, 8.2, 8.3, 8.4, 9.1, 13.3, 16.1, 17.1*, 17.4, 18.6, 18.9, 21.2, 21.4, 22.3	2.4, 3.2, 3.3, 3.6, 5.1*, 6.3, 6.8, 8.6, 8.7, 9.2, 10.3, 10.4, 11.4, 11.5, 12.5, 14.2*, 16.2*, 16.6

* strains selected to test antimicrobial activity of bovine lactoferrin.

Strains were classified as sensitive, resistant, or intermediate, according to CLSI criteria.

The Minimum Inhibitory Concentration (MIC) of MTbLF was determined following the macro dilution broth method suggested by the Clinical and Laboratory Standards Institute (CLSI) with some modifications. To perform the test, one single colony of each tested clinical isolate, grown on GVSM medium was inoculated in a 5 ml tube of PMD high iron medium and incubated under anaerobic conditions at 37°C for 24–48 h. Cultures were centrifuged at 10000 rpm for 15 min, the broth medium was poured off and cells were suspended in 5 ml of PMD medium and incubated for 2 h under anaerobic conditions at 37°C. *Gardnerella* cells were standardized to cell densities ranging from 7 log to 3 log cfu/ml then separately inoculated in 5 ml of PMD-FAC medium containing different concentrations of MTbLF (32, 16, 8, 4, 2, 1, and 0.5 mg/ml). MTbLF was provided by Metrodora Therapeutics (Brooklyn, NY) as a lyophilized powder containing >95% pure bLF at approximately 15% Fe saturation. Thus, 1 mg of MTbLF contains approximately 0.2 μg of Fe. MTbLF was dissolved to 100 mg/ml then further diluted in PMD. One tube without MTbLF was used as the positive control, whereas another without MTbLF and *Gardnerella* cells was used as medium sterility control. The antimicrobial activity of the different MTbLF concentrations was evaluated after incubation at 37°C for 24, 48, 72, and 96 h by plate count. Susceptibility was defined as the ability of MTbLF to inhibit bacterial growth by at least 50% after incubation. The inhibition rate exhibited by MTbLF was calculated after counting viable cells on GVSM agar. Each assay was performed in triplicate.

### Antibiotic interference/synergy test

Antagonistic or synergistic effect of both antibiotics and MTbLF was performed in broth culture following the NCCLS method (1987, 1993). In detail, for each tested strain, clindamycin was tested at a concentration equal to the MIC value alone and in combination with different MTbLF concentrations (8 and 4 mg/ml). Based on the MIC value exhibited by the tested strains, the antagonistic or synergistic effect of metronidazole and MTbLF was evaluated using the aforementioned MTbLF concentrations and setting the antibiotic concentration at 256 μg/ml.

### Statistical analysis

Data normality was checked by Shapiro–Wilk’s method. The homogeneity of variance of normal data was verified by the F-test. The One-way ANOVA test was used to verify if the inhibition rate displayed by the tested strains was MTbLF-or strain-specific. Pairwise comparisons between MTbLF concentrations were performed using the Tukey Honest Significant Differences test. Strain-independent difference between MTbLF concentrations was tested by Wilcoxon rank-sum test with continuity correction. A Two-tailed Student’s t-test was applied to check the antagonistic or synergistic effect of both antimicrobials and MTbLF.

All statistical tests and plots were performed and drawn using R ver. 3.6.

## Results

### Antimicrobials susceptibility test

Table 2 shows the results of antimicrobial susceptibility of the tested presumptive *G. vaginalis* clinical isolates strains. Overall, all the tested strains (71) showed resistance to metronidazole. Different susceptibility profiles were detected for clindamycin. In particular, the vast majority of the tested strains (45%), exhibiting MIC lower than 2 μg/ml, were considered sensitive. Eighteen strains (25%) with MIC higher or equal to 8 μg/ml, were classified as resistant, whereas the remaining 21 (30%) were classified as intermediate (Table 2).

**Table 2:** Susceptibility profiles of *G. vaginalis* isolates to clindamycin and metronidazole classified according to the interpretation criteria suggested by the Clinical and Laboratory Standards Institute (CSLI).

Tested antimicrobials	Interpretation criteria according to the CLSI		
	*S*	*I*	*R*
Clindamycin	45% (32/71)	30% (21/71)	25% (18/71)
Metronidazole	0% (0/71)	0% (0/71)	100% (71/71)

S, sensitive; I, intermediate; R, resistant.

### Antimicrobial activity of bovine lactoferrin against *Gardnerella* spp. isolates

The antimicrobial activity of MTbLF was tested against the presumptive *G. vaginalis* 5.1, 9.4, 13.6, 14.2, 16.2, and 17.1 strains, based on their different susceptibility profiles to clindamycin (resistance, sensitivity, and intermediate resistance).

Supplementary Figure 1 (panels a–h) shows the effect, over time (from 0 h to 96 h), of different MTbLF concentrations (32, 16, 8, 4, 2, 1, 0.5, and 0 mg/ml) against the tested presumptive *Gardnerella vaginalis* strains at different initial cell densities (from 7 to 3 log units). Overall, for all the tested strains, an MTbLF dose-dependent reduction of viable cells was achieved. In addition, the inhibitory effect exhibited by MTbLF was dependent on the time of incubation.

**Figure 1:**
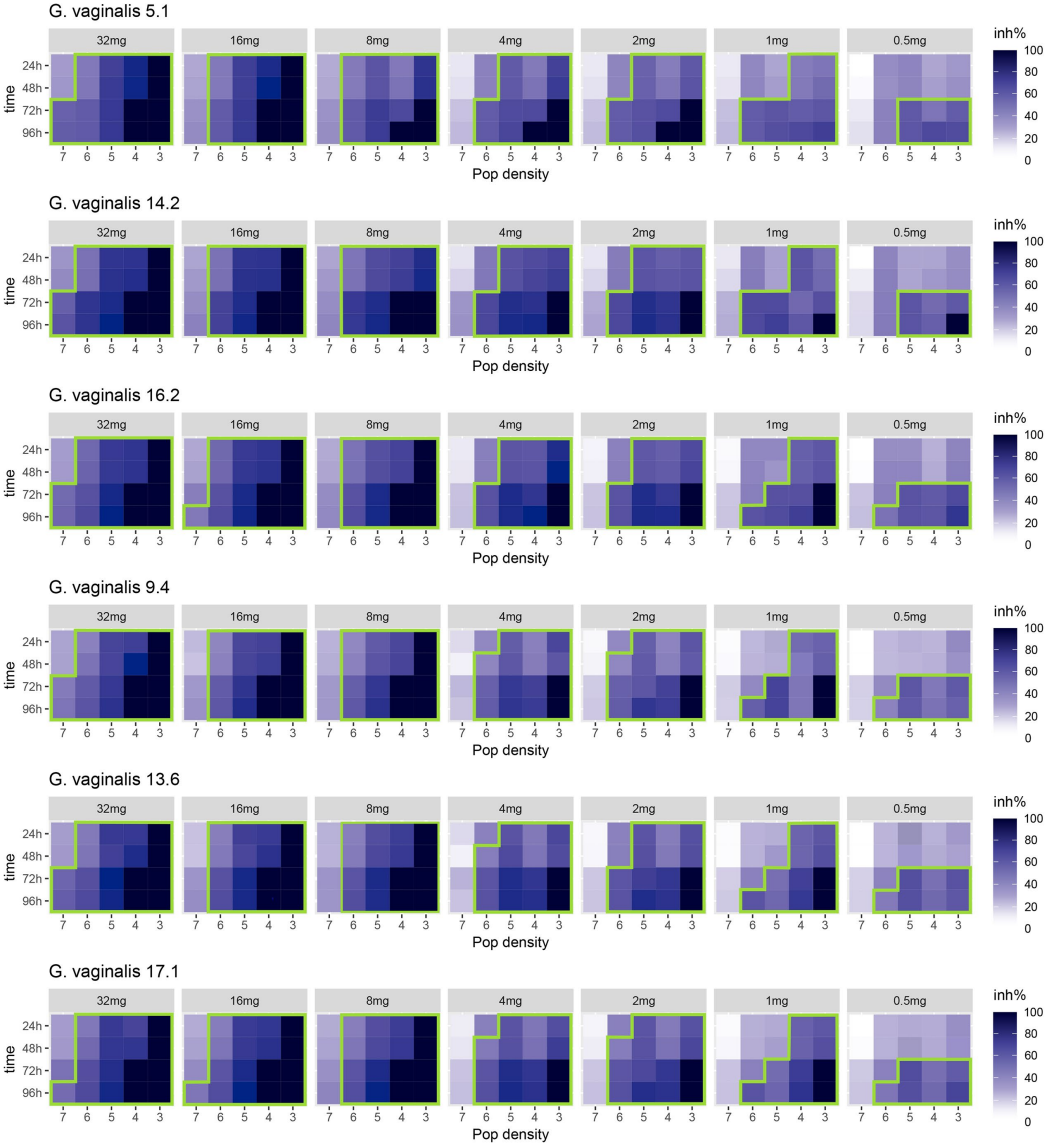
Heatmap of the inhibition rate percentages (inh%) displayed over time (from 0 h to 96 h) by presumptive *Gardnerella vaginalis* 5.1, 14.2, 16.2, 9.4, 13.6, 17.1 clinical isolates, tested at different initial cell densities (from 7 log to 3 log units), after treatment with different concentrations (32, 16, 8, 4, 2, 1, and 0.5 mg/ml) of bovine lactoferrin (MTbLF). The colours of the scale bar represent the inhibition rate percentage (inh%) in the range from 0 to 100%. The green line surrounds the inhibition rate percentages (inh%) equal to or greater than 50%.

Figure 1 shows the heat map of the inhibition rate percentage displayed by the tested strains after the treatment with different concentrations of MTbLF. Overall, similar behavior was displayed by the tested presumptive *G. vaginalis* strains with few exceptions. In fact, when the strains were standardized to cell density equal or lower to 6 log units, inhibition rates greater than 50% were recorded after 24 h of incubation in the presence of 32, 16, and 8 mg/ml of MTbLF. At MTbLF concentrations lower than 8 mg/ml, a prolonged incubation time was required to obtain inhibition rates greater than 50% (Figure 1).

MTbLF inhibited the growth of the tested presumptive *G. vaginalis* strains in a dose-dependent manner [One-way ANOVA test, *F*(6,833) = 32.09, *p* < 2e-16], but not in a strain-dependent way [*F*(5,834) = 0.15, *p* = 0.98]. In addition, Pairwise comparison performed by Tukey Honest Significant Differences test (Supplementary Figure 2) and Wilcoxon rank-sum test with continuity correction (Supplementary Figure 3A–C) suggested that MTbLF, at concentrations ranging from 32 to 8 mg/ml, exerted a statistically different antimicrobial activity compared with lower concentrations [Wilcoxon rank-sum test with continuity correction, maximum Fold inhibition Change (FC) = 1.32, W = 124,248, *p* < 2.2e-16].

### Ability of *Gardnerella* spp. isolates to use MTbLF as iron source

**Figure 2:**
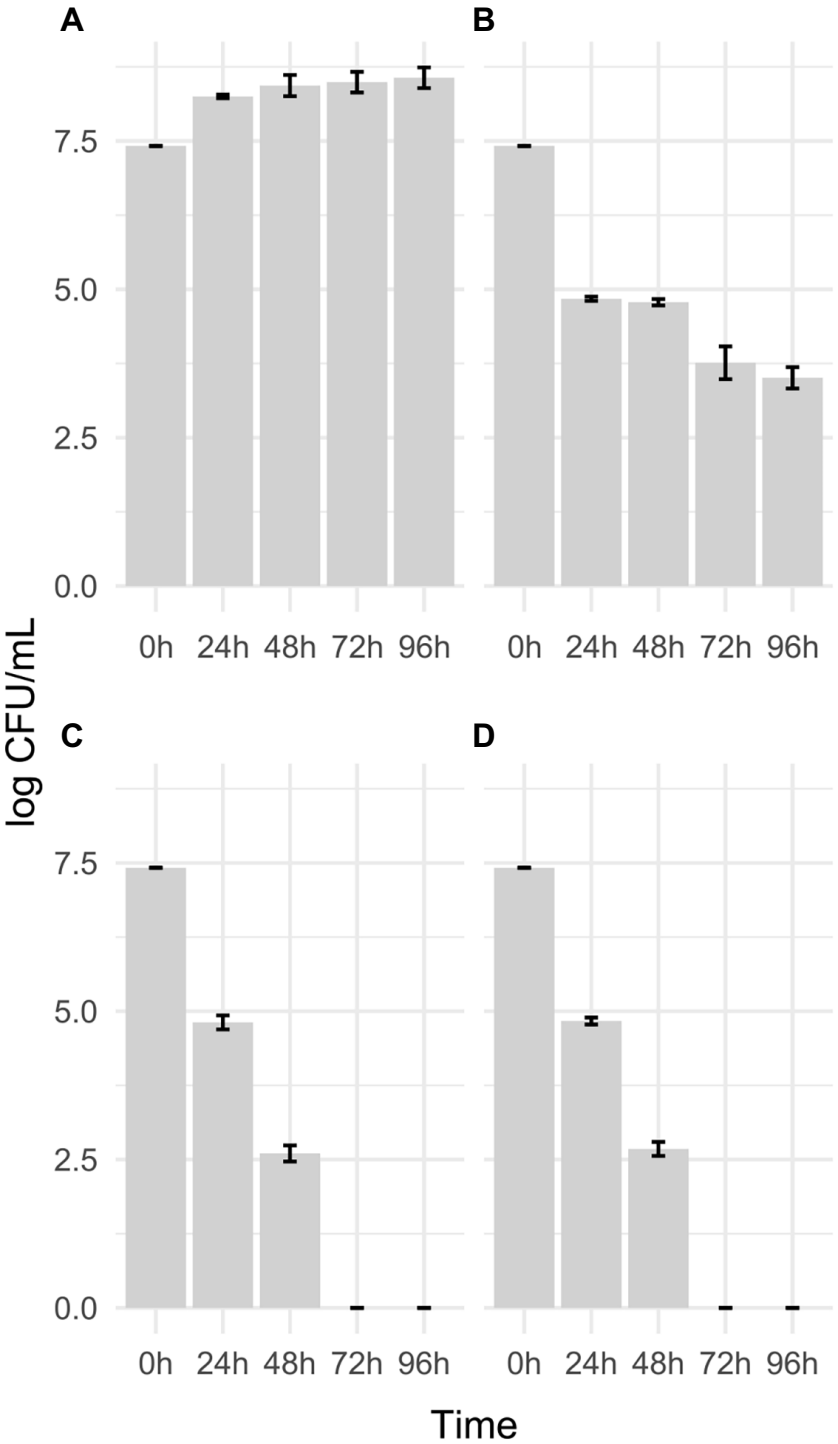
Growth performance of the presumptive *G. vaginalis* 5.1, 9.4, 14.2, and 17.1 clinical isolates in PMD medium supplemented with 10 μg/ml FAC **(A)**; 10 μg/ml FAC and 32 mg/ml MTbLF **(B)**; 32 mg/ml MTbLF **(C)** and without supplements (FAC or MTbLF; **D**).

Since *G. vaginalis* expresses hLf binding proteins (Jarosik and Land, 2000), the possibility that bovine lactoferrin could serve as an iron source was tested. Figure 2 shows the growth performance of the presumptive *G. vaginalis* 5.1, 9.4, 14.2, and 17.1 strains in PMD medium without supplements (FAC or MTbLF) and in PMD medium supplemented with 10 μg/ml FAC; or 10 μg/ml FAC and 32 mg/ml MTbLF; or 32 mg/ml MTbLF alone. Overall, all the tested strains were able to grow only in PMD medium supplemented with 10 μg/ml of FAC, which corresponds to 2 μg/ml elemental iron. Differently, in PMD medium without supplements (FAC or MTbLF) and in PMD medium supplemented with 32 mg/ml MTbLF (corresponding to 7 μg/ml elemental iron in the form of Fe-bLF), a reduction of viable cells of about 2.4 log units and 4.8 log units was observed after 24 and 48 h of incubation, respectively (Figure 2). In addition, after 72 and 96 h of incubation the number of viable cells were below the limit of detection (<10 cfu/ml).

### Antibiotic interference/synergy test

**Figure 3:**
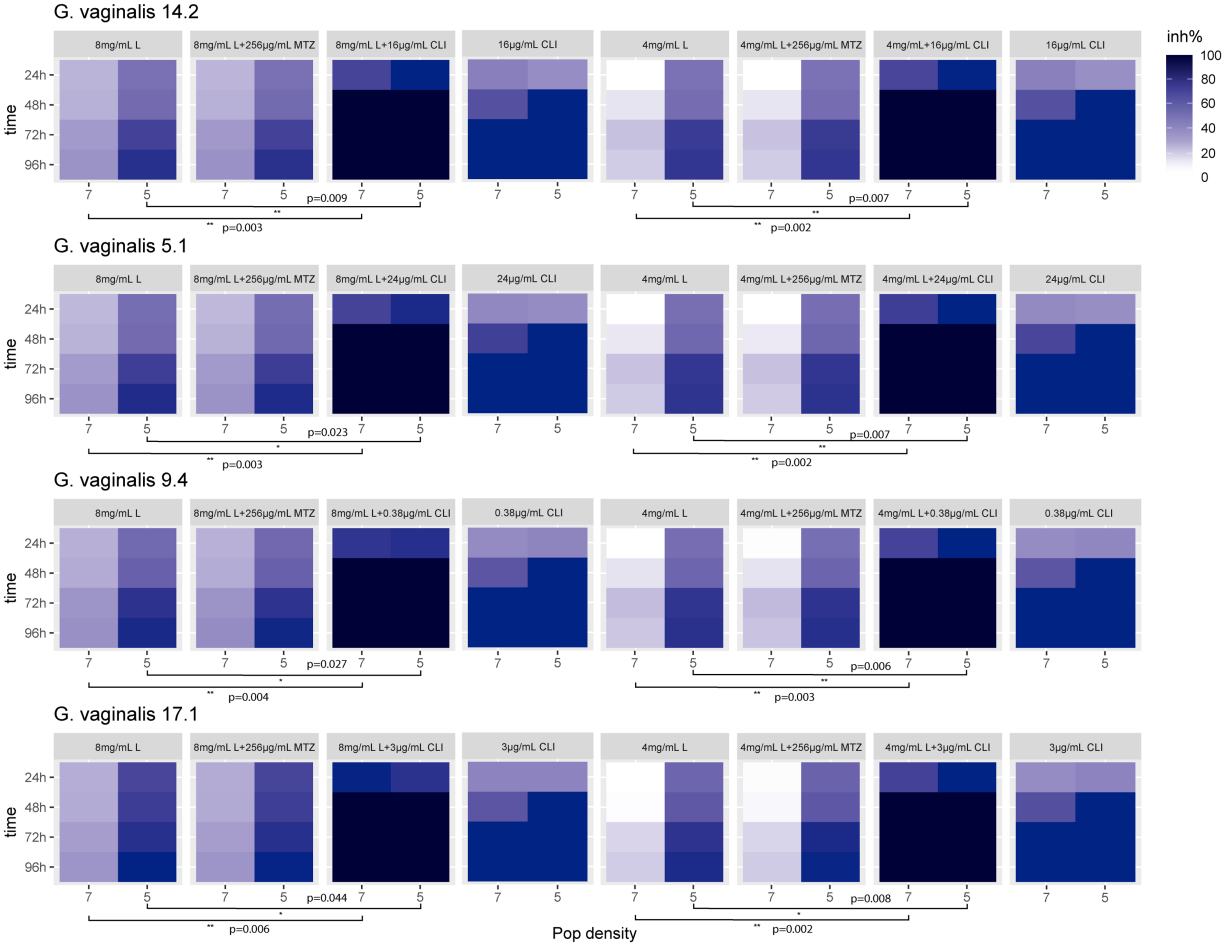
Heat map of the inhibition rate percentage (inh%) exhibited by the presumptive *G. vaginalis* 14.2, 5.1, 9.4, and 17.1 clinical isolates, tested at both 7 and 5 log units, in the presence of bovine lactoferrin (MTbLF; 8 or 4 mg/ml) and antibiotic (metronidazole or clindamycin). Stars indicate statistically significant differences based on two-tailed Student’s *t*-test.

Figure 3 shows the inhibition percentage (inh%) exhibited by the presumptive *G. vaginalis* 5.1, 9.4, 14.2, and 17.1 strains, standardized to both 7 and 5 log units, in the presence of both MTbLF (8 or 4 mg/ml) and antibiotic (metronidazole or clindamycin at a concentration equal to the MIC value). For all the tested strains, the combination MTbLF and metronidazole (256 μg/ml) did not provide a synergistic effect. In fact, as shown by T-test, no statistically significant differences were observed among the inhibition rate observed after MTbLF treatment with or without metronidazole. Differently, based on the *T*-test (Figure 3), a statistically significant improvement of the inhibition rate of clindamycin and MTbLF was revealed for all the tested strains.

## Discussion

*Gardnerella vaginalis* plays a key role in the pathogenesis of bacterial vaginosis (BV; Gilbert et al., 2013; Machado and Cerca, 2015; Roselletti et al., 2020). Currently, the standard-of-care therapeutic approach for BV treatment is based on antibiotic administration, often metronidazole and/or clindamycin (Machado et al., 2016; Mendling et al., 2016; Verstraelen and Swidsinski, 2019). Although the antibiotic treatment is effective in about 80% of BV cases, a high recurrence rate (>50%) is reported within 6–12 months (Bradshaw et al., 2006a). In addition, prolonged and repeated antibiotic treatments facilitate the development of resistant pathogens (Beigi et al., 2004; Ferris et al., 2004; Bradshaw et al., 2006b). In this context, resistance to both metronidazole and clindamycin is well documented among *G. vaginalis* isolates (Knupp de Souza et al., 2016; Schuyler et al., 2016; Hopp et al., 2022), therefore complementary and/or alternative therapeutic approaches are needed.

The present study evaluated the susceptibility of 71 presumptive *G. vaginalis* clinical isolates to metronidazole and clindamycin and investigated the *in vitro* antimicrobial activity of bovine lactoferrin (MTbLF) at different concentrations. The possibility that MTbLF could serve as an iron source for *G. vaginalis* growth was evaluated. In addition, the antagonistic or synergistic effect of MTbLF and antimicrobials against resistant, intermediate, and sensitive presumptive *G. vaginalis* clinical isolates, were also investigated. The concentrations of iron, used in the present study, are in the range of iron levels measured in vaginal fluid (0.1–4 μg/ml; unpublished data) and concentrations of MTbLF used in cell culture are achieved and maintained in vaginal fluid following vaginal administration of MTbLF (Hopp et al., 2022).

Corroborating previously reported data (Petrina et al., 2017; Li et al., 2020), the present study highlighted a high rate of antibiotic resistance among the tested clinical isolates. In fact, all the tested strains exhibited resistance to metronidazole with MIC values higher than 256 μg/ml. Similarly, Schuyler and co-workers (2016), setting 32 μg/ml as a breakpoint to discriminate among metronidazole sensitive and resistant strains, showed that all the *G. vaginalis* strains allocated to clades 3 and 4 were resistant to metronidazole. A high percentage of metronidazole resistance was also found by Knupp de Souza et al. (2016) which evaluated the antimicrobial susceptibility patterns of *G. vaginalis* clinical isolates against antimicrobial drugs of clinical interest for the BV treatment. In particular, among the 204 *G. vaginalis* isolates, 59.8% of them were classified as resistant. Similarly, Austin et al. (2006) observed that 68% of the tested *G. vaginalis* strains exhibited resistance to metronidazole with MIC higher than 256 μg/ml. These data could be explained considering that facultative anaerobic bacteria are generally not inhibited by nitroimidazoles since the nitro group reduction depends on the absence of oxygen (Haggoud et al., 1994). In addition, as explained by McLean and McGroaty (1996), the density of an outer surface fibrillar layer, consisting of fimbriae, can influence the susceptibility of *G. vaginalis* strains to metronidazole. In particular, the authors suggested that the presence of a dense layer, by impeding the penetration of metronidazole, is related to less sensitivity to this antimicrobial. Therefore, the high resistance rate observed among clinical isolates could be due to the exposure to selective pressures, such as the *in vivo* exposure to metronidazole, which induces the development of a degree of resistance to this antimicrobial drug.

Clindamycin is able to exert anti-inflammatory properties and possess a broad range of activity against BV associated bacteria (Ianaro et al., 2020). In the present study, the vast majority of the tested clinical isolates were classified as clindamycin sensitive or intermediate. The low resistance rate to clindamycin was recently reported by Li and co-workers (2020) which, investigating the susceptibility of planktonic *G. vaginalis* and biofilms to metronidazole and clindamycin, observed high susceptibility rate to clindamycin at both planktonic and biofilm levels (Li et al., 2020).

It is well known that bovine lactoferrin has antimicrobial, antiviral, and antifungal properties (Jenssen and Hancock, 2009). In particular, the antimicrobial activity is related to the ability to sequester iron, which is essential for the growth of almost all microorganisms, including those belonging to the *G. vaginalis* species (Jarosik et al., 1998). In addition, lactoferrin exerts a direct action on the outer membrane of Gram-negative bacteria, increasing permeability (Ellison et al., 1988), interfering with flagellar motility, and is able to antagonize pathogens through the prevention of both bacterial adhesion and invasion (Superti et al., 2008). Previous studies demonstrated its antimicrobial effect against bacteria involved in vaginal infections such as *Chlamydia trachomatis* (Sessa et al., 2017) and *Staphylococcus aureus* (Naidu et al., 1991). In addition, Wakabayashi and co-workers (1996) suggested that bovine lactoferrin and bovine lactoferrin-derived peptides cooperatively act with azole against *Candida albicans* (Wakabayashi et al., 1996; Cianci et al., 2020). Although the ability of lactoferrin to counteract bacterial and fungal vaginal infections clearly emerged, little is known about the antimicrobial effect of bovine lactoferrin against *Gadnerella* spp.

The present study examined the ability of MTbLF to *in vitro* inhibit the growth of presumptive *G. vaginalis* clinical isolates. Results revealed that the antimicrobial effect was dose-dependent and not strain-dependent, suggesting the ability of the tested strains to utilize the iron for growth. *G. vaginalis* strains can utilize a number of different iron containing compounds, including iron salts, hemin, hemoglobin, and human lactoferrin but not bovine lactoferrin (Jarosik et al., 1998). The report that bovine lactoferrin does not support *G. vaginalis* growth was confirmed herein with multiple presumptive clinical isolates. The specificity of *G. vaginalis* lactoferrin binding proteins for human lactoferrin (Jarosik and Land, 2000) suggests BV therapeutics employing bovine lactoferrin are preferable to recombinant human lactoferrin. In addition, when MTbLF was used in combination with clindamycin, a synergistic effect was detected. This evidence suggests that MTbLF could improve the effectiveness of traditional antimicrobial, contributing to hindering antimicrobial resistance. Our data align with previous studies, emphasizing that the use of natural bioactive substances, such as lactoferrin, can represent an innovative strategy to treat microbial infections and tackle antibiotic resistance (Ciccaglione et al., 2019). The ability of bovine lactoferrin to potentiate the effect of antibiotics was previously described for the treatment of *Helicobacter pylori* infection (Di Mario et al., 2003; de Bortoli et al., 2007). In particular, testing *Helicobacter pylori* strains resistant to levofloxacin, bovine lactoferrin was able to potentiate the effect of levofloxacin, reducing the level of resistance of the bacterium and, thereby, increasing the overall efficacy of the treatment (Ciccaglione et al., 2019).

In the present study clinical isolates were identified as presumptive *G. vaginalis* stains through species specific PCR. Further studies are needed to confirm the taxonomy affiliation of the strains in order to confirm the high *G. vaginalis* species occurrence in BV. A limitation of this study is that our *in-vitro* work does not simulate the polymicrobial environment seen in BV and our ability to precisely control iron in culture media is not fully representative of the nutritional environment of the vagina, which is in constant flux. The vaginal environment contains multiple source of iron including blood, heme, transferrin and bacterial siderophores that may have impact on overall iron availability to a given bacterial species. Furthermore, the well know presence of biofilm in BV may also complicate the local bioavailability of lactoferrin. Nonetheless, we believe the ability of lactoferrin to suppress *Gardnerella* species and act synergistically with antibiotics, albeit in a tightly controlled *in-vitro* environment, provides an insight that warrants further study. The safety and vaginal pharmacokinetics of multiple formulations of MTbLF are under evaluation in a Phase 1 clinical trial (ACTRN12619000295145). Characterization of lactoferrin and lactoferrin fragments in vaginal fluid specimens from that study demonstrate that iron-sequestering capacity can be maintained with vaginally administered lactoferrin. Additional trials to evaluate the effect of MTbLF on the treatment and prevention of recurrence of BV are planned.

## Data availability statement

The datasets presented in this study can be found in online repositories. The names of the repository/repositories and accession number(s) can be found in the article/Supplementary material.

## Author contributions

AP, M-AM, CR, and GAG designed the experiment. AP performed the experiments. TM and SC analyzed the data. AP and TM wrote the first draft of the manuscript. M-AM, CR, CC, and GAG revised the manuscript. All authors contributed to the article and approved the submitted version.

## Funding

The present study was funded and commissioned by Metrodora Therapeutics (Brooklyn, NY). Project title: Characterization of the antimicrobial activity of bovine lactoferrin; use of bovine lactoferrin for microbiome modulation and the treatment of infection including genitounrinary infection and preferably bacterial vaginosis. Project number: 5A762192028.

## Conflict of interest

The authors declare that the research was conducted in the absence of any commercial or financial relationships that could be construed as a potential conflict of interest.

AP, CC, and CLR declare that they are members of ProBioEtna, a spinoff of the University of Catania, Italy. In addition, the authors declare that they do not have any personal, financial, professional, political, or legal interests with a significant chance of interfering with the performance of their ethical or legal duties.

GAG owns equity and authors M-AHM and GAG are employed by Metrodora Therapeutics LLC, Brooklyn, NY. This study received funding from Metrodora Therapeutics LLC, Brooklyn, NY. The funder had the following involvement with the study design, interpretation of data and editing the article.

## Publisher’s note

All claims expressed in this article are solely those of the authors and do not necessarily represent those of their affiliated organizations, or those of the publisher, the editors and the reviewers. Any product that may be evaluated in this article, or claim that may be made by its manufacturer, is not guaranteed or endorsed by the publisher.
