# Supplementary figures and images for: Antimicrobial activity of bovine lactoferrin against Gardnerella species clinical isolates
Source: Front Microbiol. 2022 Sep 8;13:1000822. doi: 10.3389/fmicb.2022.1000822 (PMC9678186; doi:10.3389/fmicb.2022.1000822)

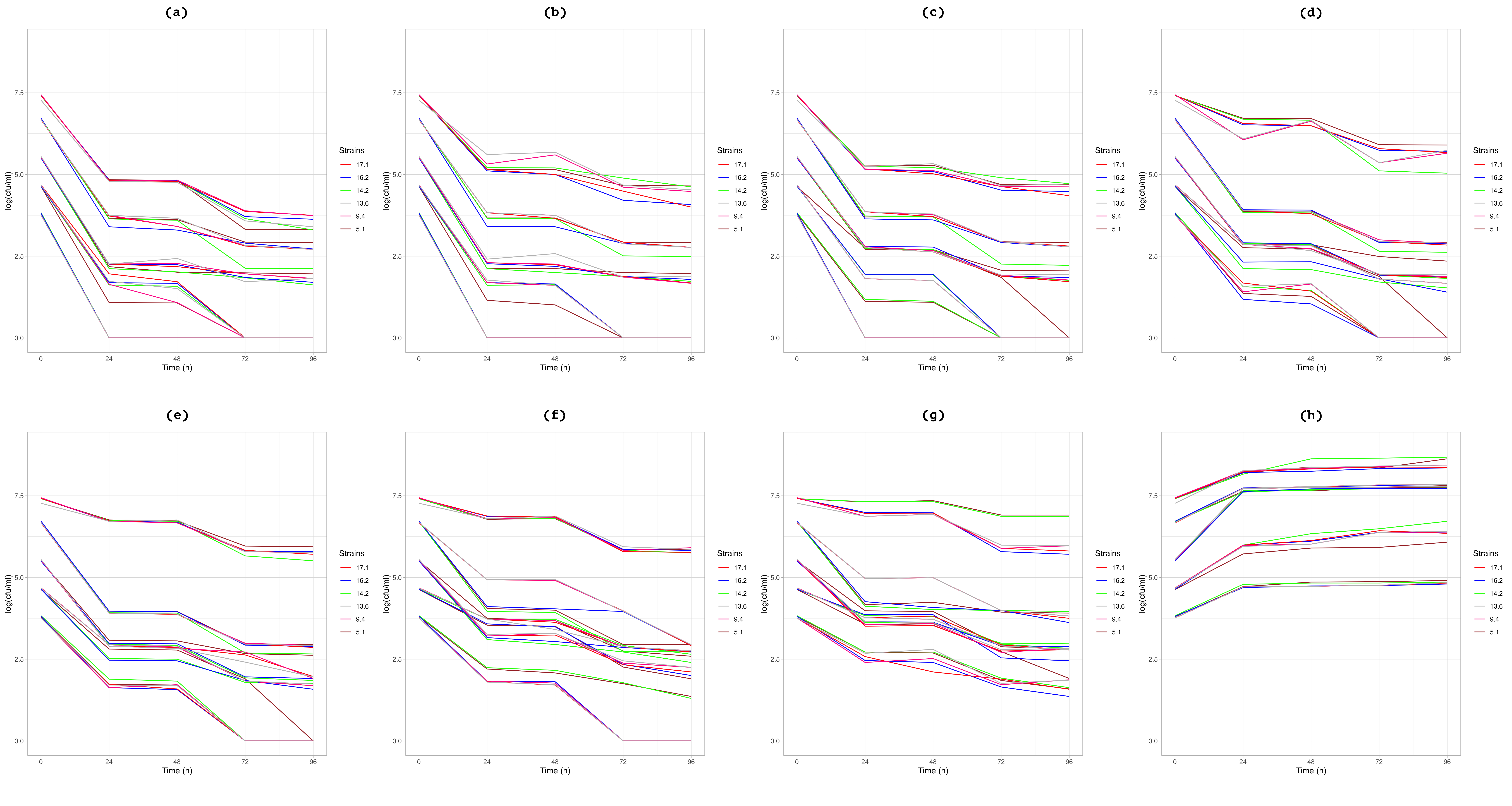

Supplement: Supplementary file 1 [file Image_1.TIF]

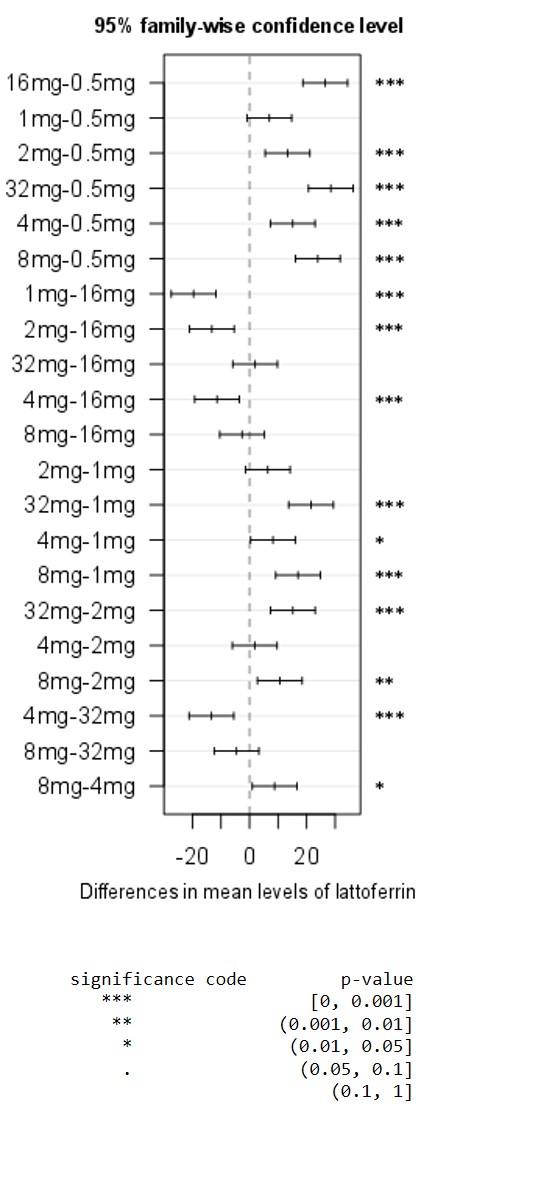

Supplement: Supplementary file 2 [file Image_2.JPEG]

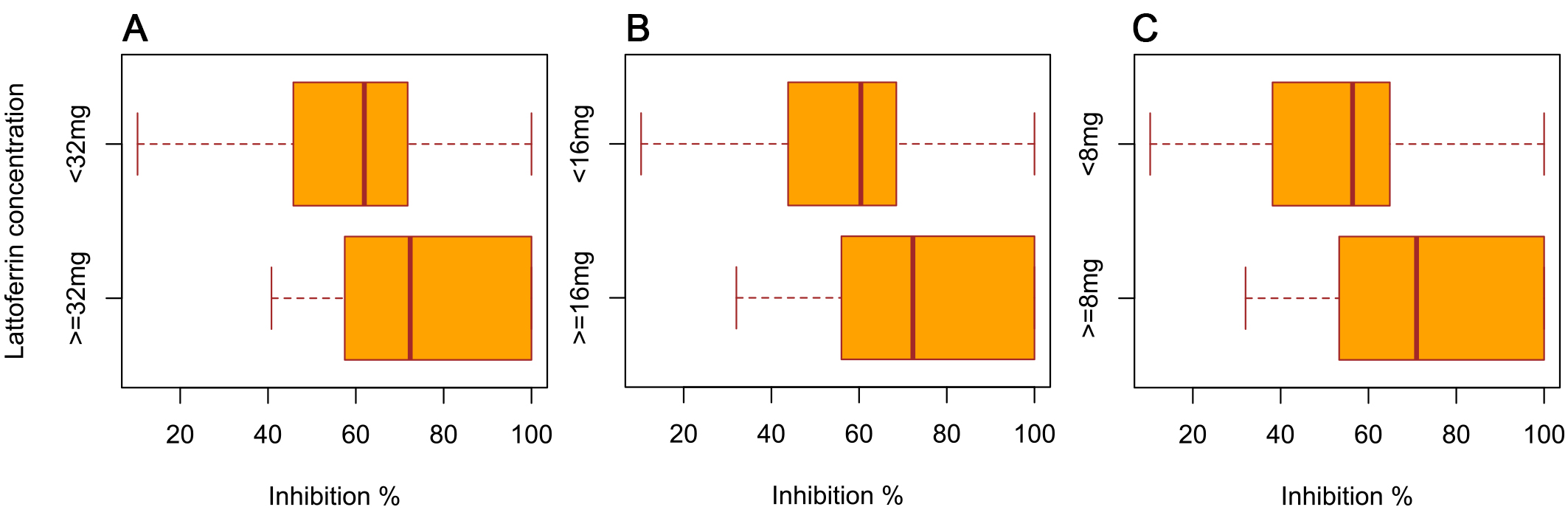

Supplement: Supplementary file 3 [file Image_3.JPEG]
