# Supplementary material for: Antimicrobial activity of bovine lactoferrin against Gardnerella species clinical isolates
Source: Front Microbiol. 2022 Sep 8;13:1000822. doi: 10.3389/fmicb.2022.1000822 (PMC9678186; doi:10.3389/fmicb.2022.1000822)
Supplement: Supplementary file 4 [file Table_1.DOCX]

**Antimicrobial activity of bovine lactoferrin against *Gardnerella* species clinical isolates**

**Alessandra Pino^1,2^, Tommaso Mazza^3^, Maura-Ann H. Matthews^4^, Stefano Castellana^3^, Cinzia Caggia^1,2^, Cinzia L. Randazzo^1,2*^, Gary A. Gelbfish^4,5^**

^1^Department of Agricultural, Food and Environment, University of Catania, Santa Sofia street, 100, 95123 Catania, (Italy);

^2^ProBioEtna srl, Spin-off of University of Catania, Santa Sofia street, 100, 95123 Catania, (Italy);

^3^ Bioinformatics unit, Fondazione IRCCS Casa Sollievo della Sofferenza Viale Capuccini 1, 71013 San Giovanni Rotondo (FG), Italy;

^4^Metrodora Therapeutics LLC, 2502 Avenue I, Brooklyn, NY, (USA);

^5^Department of Surgery, Mount Sinai School of Medicine, New York, NY (USA).

*** Correspondence:**Cinzia Lucia Randazzo
[cranda@unict.it](mailto:cranda@unict.it)

Supplementary Material

## Supplementary Figures

**Supplementary Figure 1.** Effect, over time (from 0h to 96h), of different bovine lactoferrin (MTbLF) concentrations (a: 32 mg/ml, b: 16 mg/ml, c: 8 mg/ml, d: 4 mg/ml, e: 2 mg/ml, f: 1 mg/ml, g: 0.5 mg/ml, and h: 0 mg/ml) against the presumptive *Gardnerella vaginalis* 5.1, 14.2, 16.2, 17.1, 9.4, and 13.6 clinical isolates at different initial cell densities (from 7 log to 3 log units).

**Supplementary Figure 2.** Pairwise comparison, performed by Tukey Honest Significant Differences test, of the antimicrobial effect elicited by different bovine lactoferrin (MTbLF) concentrations (32 mg/ml, 16 mg/ml, 8 mg/ml, 4 mg/ml, 2 mg/ml, 1 mg/ml, 0.5 mg/ml, and 0 mg/ml) against the presumptive *Gardnerella vaginalis* 5.1, 14.2, 16.2, 17.1, 9.4, and 13.6 clinical isolates at different initial cell densities (from 7 to 3 log units).

**Supplementary Figure 3.** Distribution of inhibition rates obtained with high (=32 mg/ml, ≥16 mg/ml, or ≥8 mg/ml) versus low MTbLF concentrations; results of Wilcoxon rank-sum tests with continuity correction, and fold-change (FC) of inhibition differences between groups.

Panel A: FC (≥32 vs ≤32)=1.23; W=58865; p=8.851e-11; panel B: FC (≥16 vs ≤16)=1.27; W=100498; p=2.2e-16; panel C: FC (≥8 vs ≤8)=1.32; W=124248; p=2.2e-16.

**Supplementary Table1**. ID, isolation source and features of the presumptive *Gardnerella vaginalis* stains tested in the present study.

| Strain ID | Patients | Isolation source |
| --- | --- | --- |
| 2.4 | 2 | Vaginal discharge |
| 3.15 | 3 | Vaginal discharge |
| 3.2 | 3 | Vaginal discharge |
| 3.21 | 3 | Vaginal discharge |
| 3.3 | 3 | Vaginal discharge |
| 3.6 | 3 | Vaginal discharge |
| 3.8 | 3 | Vaginal discharge |
| 4.2 | 4 | Vaginal discharge |
| 4.3 | 4 | Vaginal discharge |
| 4.4 | 4 | Vaginal discharge |
| 4.5 | 4 | Vaginal discharge |
| 5.1 | 5 | Vaginal discharge |
| 6.1 | 6 | Vaginal discharge |
| 6.2 | 6 | Vaginal discharge |
| 6.3 | 6 | Vaginal discharge |
| 6.5 | 6 | Vaginal discharge |
| 6.8 | 6 | Vaginal discharge |
| 7.2 | 7 | Vaginal discharge |
| 7.4 | 7 | Vaginal discharge |
| 7.5 | 7 | Vaginal discharge |
| 7.9 | 7 | Vaginal discharge |
| 7.10 | 7 | Vaginal discharge |
| 7.21 | 7 | Vaginal discharge |
| 8.2 | 8 | Vaginal discharge |
| 8.3 | 8 | Vaginal discharge |
| 8.4 | 8 | Vaginal discharge |
| 8.6 | 8 | Vaginal discharge |
| 8.7 | 8 | Vaginal discharge |
| 9.1 | 9 | Vaginal discharge |
| 9.2 | 9 | Vaginal discharge |
| 9.4 | 9 | Vaginal discharge |
| 10.3 | 10 | Vaginal discharge |
| 10.4 | 10 | Vaginal discharge |
| 10.6 | 10 | Vaginal discharge |
| 10.7 | 10 | Vaginal discharge |
| 10.9 | 10 | Vaginal discharge |
| 10.13 | 10 | Vaginal discharge |
| 11.1 | 11 | Vaginal discharge |
| 11.3 | 11 | Vaginal discharge |
| 11.4 | 11 | Vaginal discharge |
| 11.5 | 11 | Vaginal discharge |
| 11.6 | 11 | Vaginal discharge |
| 12.1 | 12 | Vaginal discharge |
| 12.2 | 12 | Vaginal discharge |
| 12.5 | 12 | Vaginal discharge |
| 12.7 | 12 | Vaginal discharge |
| 12.8 | 12 | Vaginal discharge |
| 13.3 | 13 | Vaginal discharge |
| 13.6 | 13 | Vaginal discharge |
| 14.2 | 14 | Vaginal discharge |
| 14.5 | 14 | Vaginal discharge |
| 14.8 | 14 | Vaginal discharge |
| 16.1 | 16 | Vaginal discharge |
| 16.2 | 16 | Vaginal discharge |
| 16.6 | 16 | Vaginal discharge |
| 17.1 | 17 | Vaginal discharge |
| 17.4 | 17 | Vaginal discharge |
| 18.3 | 18 | Vaginal discharge |
| 18.6 | 18 | Vaginal discharge |
| 18.9 | 18 | Vaginal discharge |
| 19.6 | 19 | Vaginal discharge |
| 20.2 | 20 | Vaginal discharge |
| 20.4 | 20 | Vaginal discharge |
| 20.5 | 20 | Vaginal discharge |
| 21.2 | 21 | Vaginal discharge |
| 21.4 | 21 | Vaginal discharge |
| 22.3 | 22 | Vaginal discharge |
| 26.3 | 26 | Vaginal discharge |
| 26.7 | 26 | Vaginal discharge |
| 26.9 | 26 | Vaginal discharge |
| 27.4 | 27 | Vaginal discharge |
